# Supplementary material for: Islands of genomic stability in the face of genetically unstable metastatic cancer
Source: bioRxiv. 2024 Jan 29:2024.01.26.577508. Preprint. [Version 1] doi: 10.1101/2024.01.26.577508 (PMC10862738; doi:10.1101/2024.01.26.577508)
Supplement: Supplement 1 [file NIHPP2024.01.26.577508v1-supplement-1.pdf]

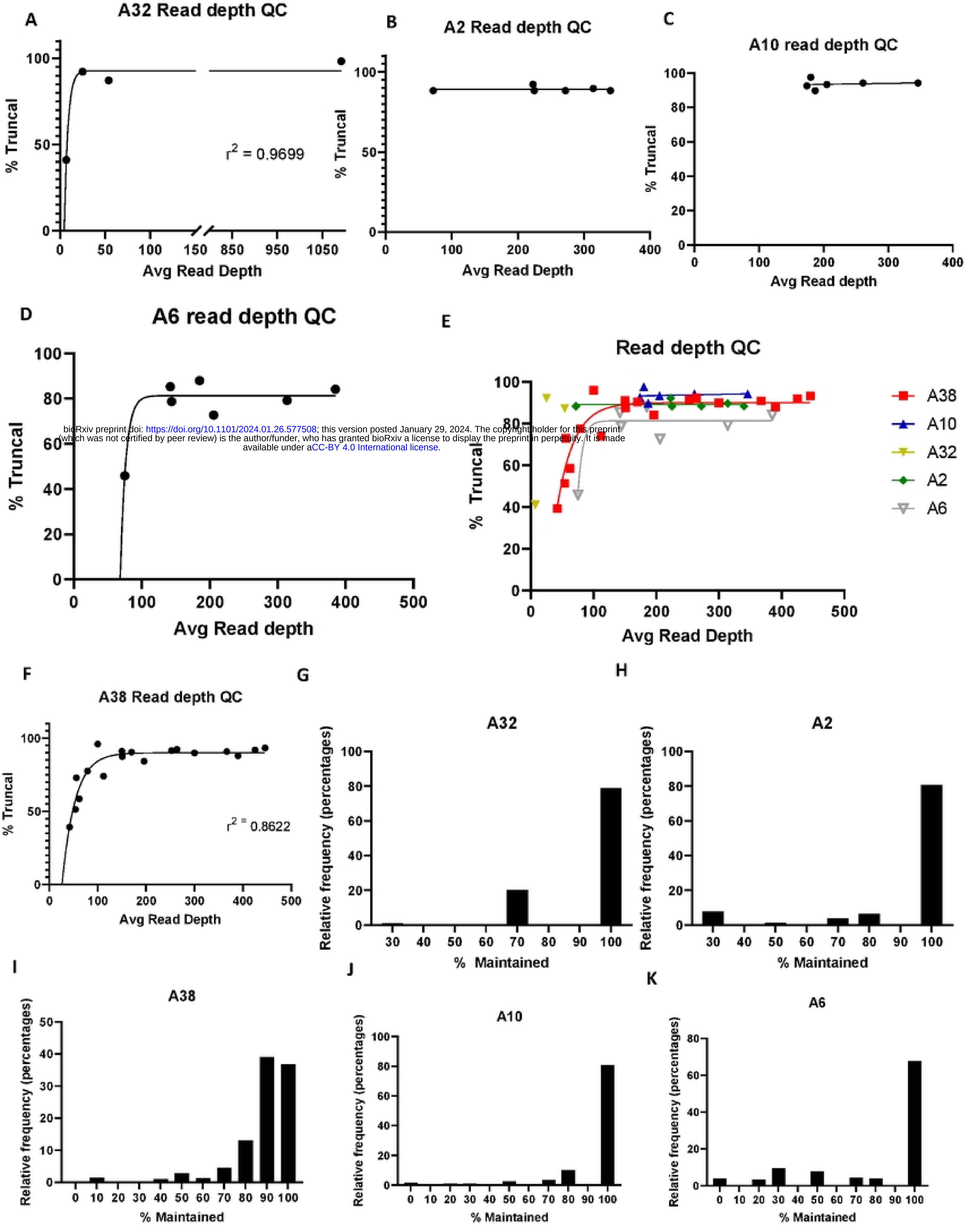

bioRxiv preprint doi: https://doi.org/10.1101/2024.01.26.577508; this version posted January 29, 2024. The copyright holder for this preprint (which was not certified by peer review) is the author/funder, who has granted bioRxiv a license to display the preprint in perpetuity. It is made available under aCC-BY 4.0 International license.

Supplemental Figure 1. Quality control analysis plotting average read depth for each sample against the calculated percent truncal for that sample, broken down by each case, and then shown overall for all cases. Histograms showing maintenance of PAMs for each case are also shown.

Supplement 2

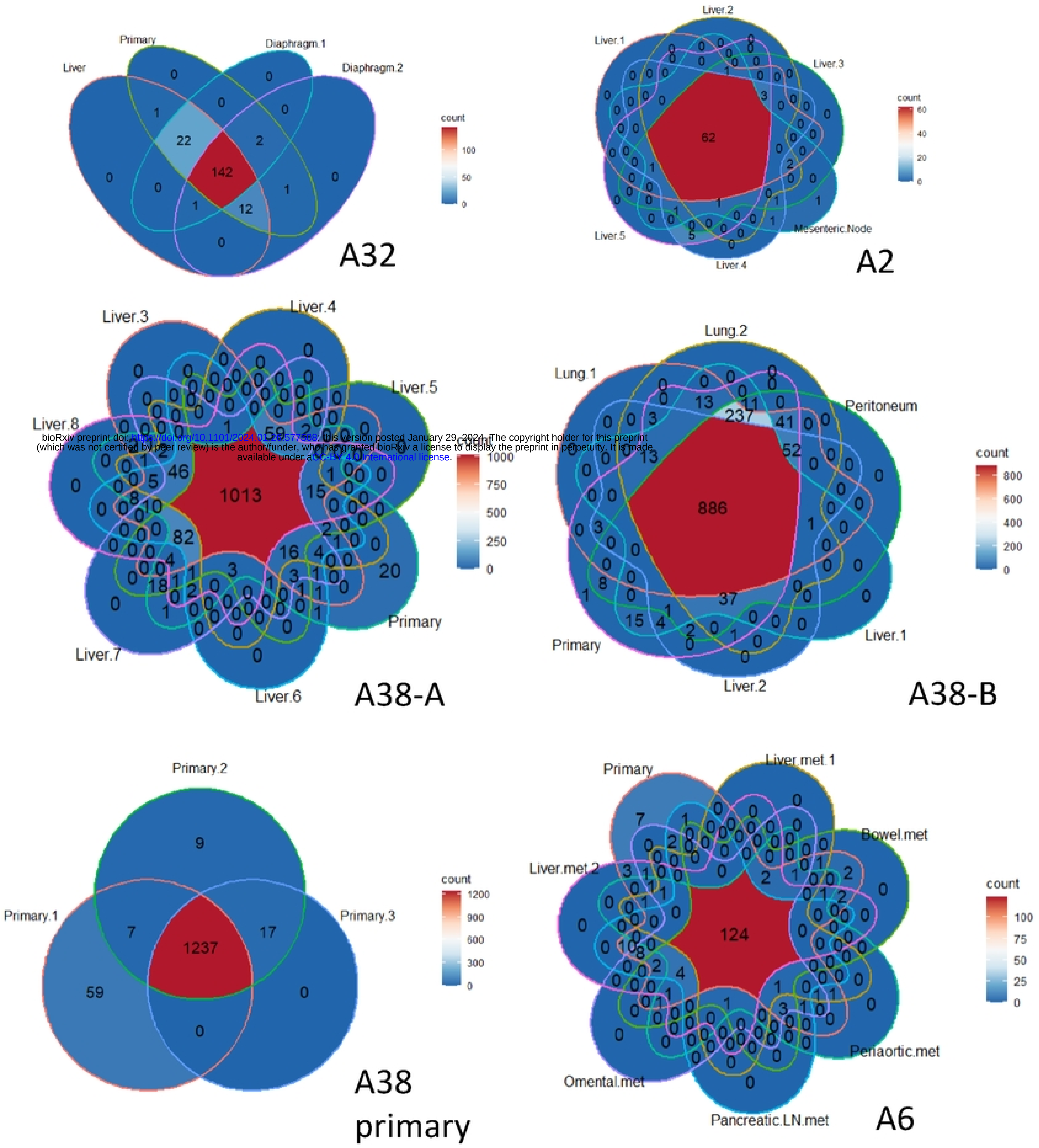

bioRxiv preprint doi: https://doi.org/10.1101/2024.01.26.577508; this version posted January 29, 2024. The copyright holder for this preprint (which was not certified by peer review) is the author/funder, who has granted bioRxiv a license to display the preprint in perpetuity. It is made available under aCC-BY 4.0 International license.

Supplemental Figure 2. Venn diagrams of PAM overlap for each case. A38 is broken into two Venn diagrams as a maximum of 7 samples can be plotted per diagram. Diagrams created with ggvenn from the gglot2 R package.

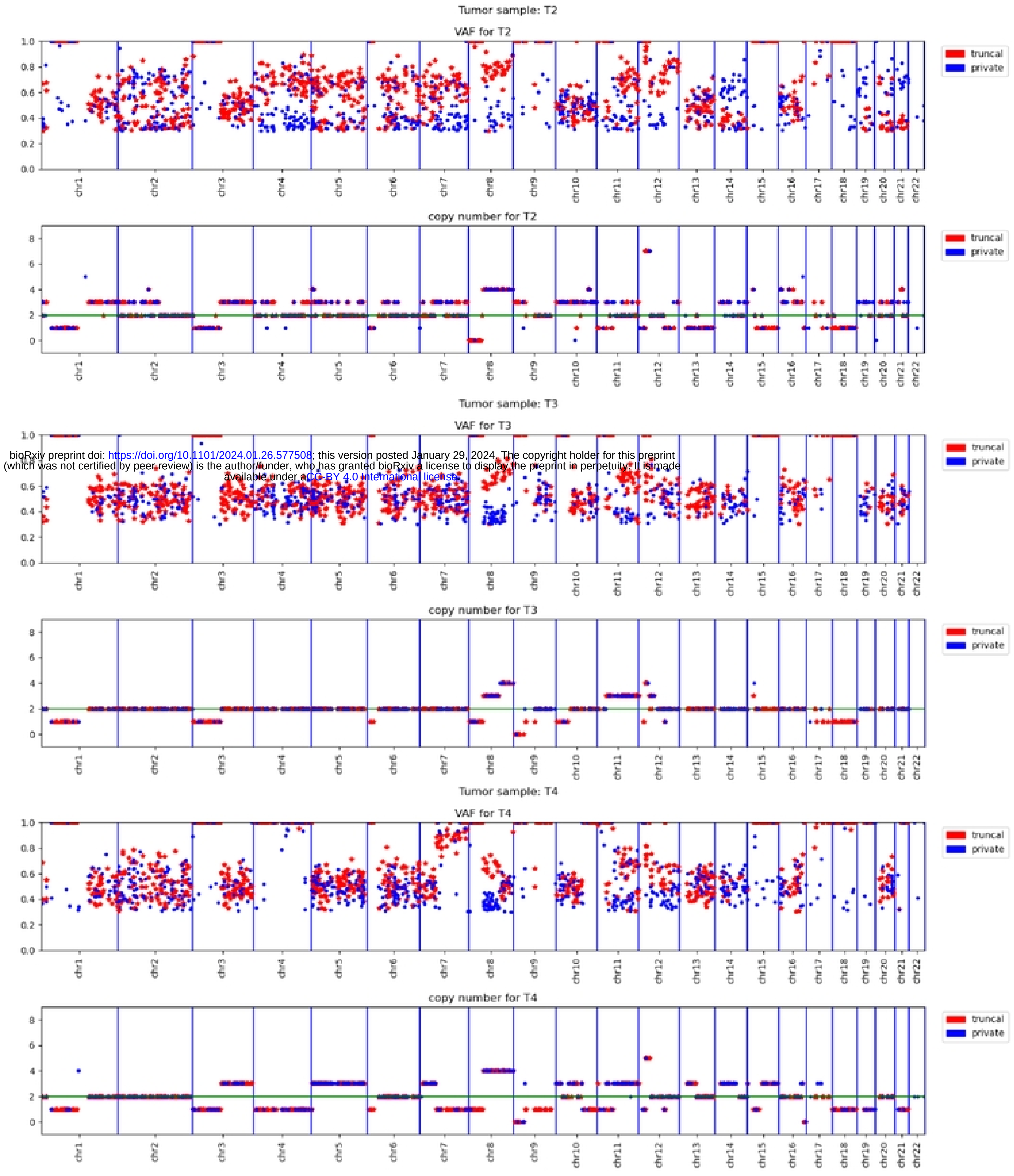

bioRxiv preprint doi: https://doi.org/10.1101/2024.01.26.577508; this version posted January 29, 2024. The copyright holder for this preprint (which was not certified by peer review) is the author/funder, who has granted bioRxiv a license to display the preprint in perpetuity. It is made available under aCC-BY 4.0 International license.

Supplemental Figure 3: VAF and CN for truncal and private PAMs for WGS of A38 cell lines T2-4.

Supplement 4

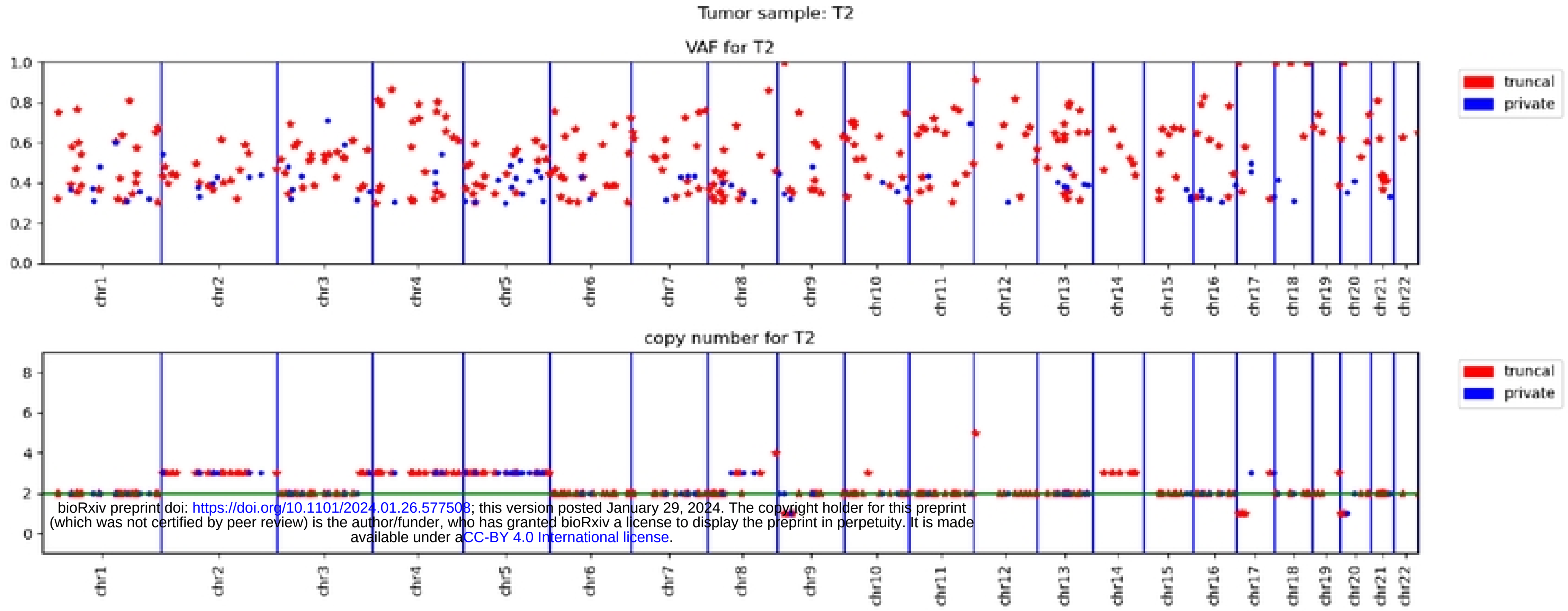

bioRxiv preprint doi: https://doi.org/10.1101/2024.01.26.577508; this version posted January 29, 2024. The copyright holder for this preprint (which was not certified by peer review) is the author/funder, who has granted bioRxiv a license to display the preprint in perpetuity. It is made available under aCC-BY 4.0 International license.

Supplemental Figure 4: VAF and CN for truncal and private PAMs for WGS of A32 cell line T2.

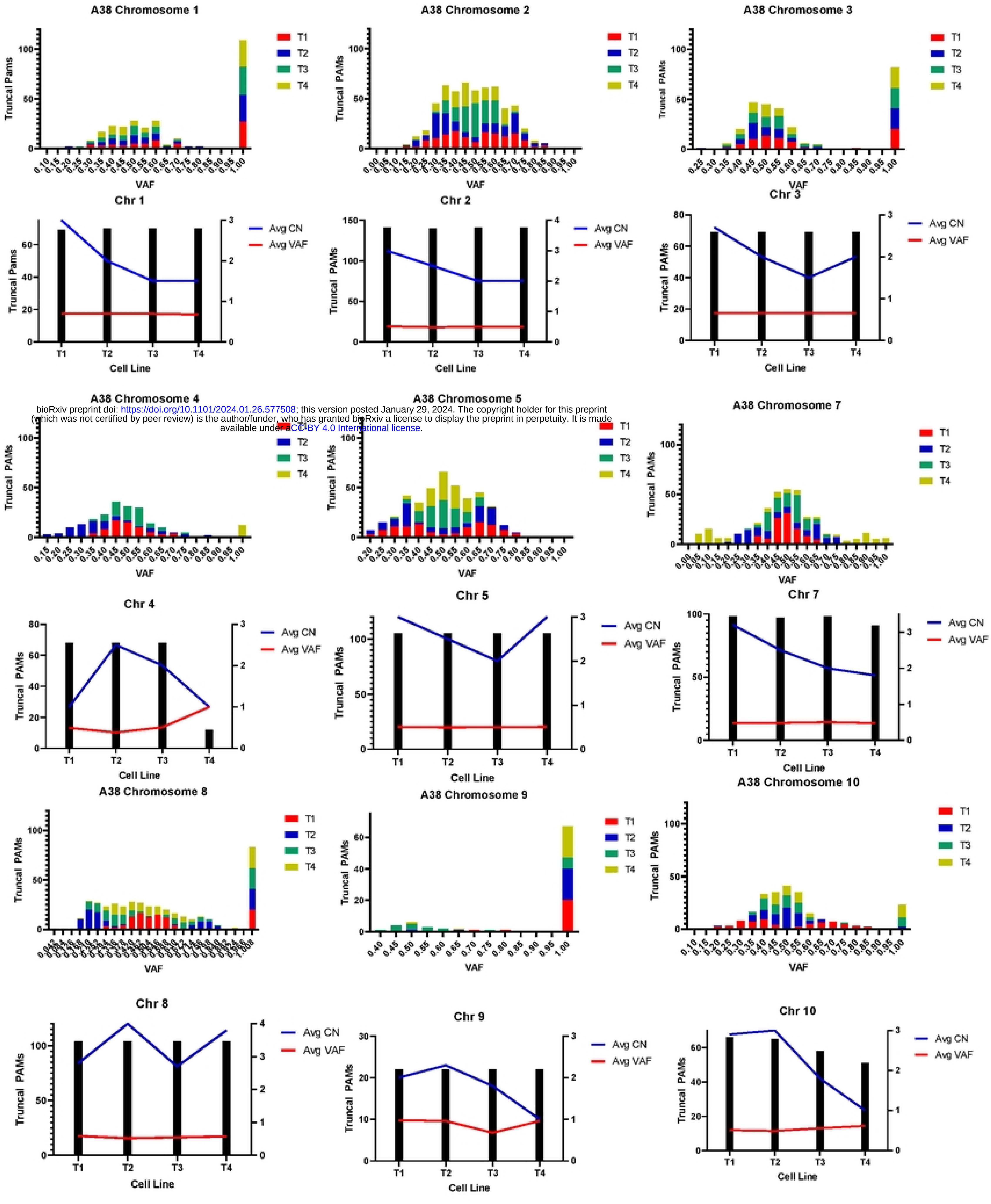

bioRxiv preprint doi: https://doi.org/10.1101/2024.01.26.577508; this version posted January 29, 2024. The copyright holder for this preprint (which was not certified by peer review) is the author/funder, who has granted bioRxiv a license to display the preprint in perpetuity. It is made available under aCC-BY 4.0 International license.

Supplemental Figure 5: Histograms and CN/VAF analysis for truncal PAMs in A38 WGS cell line data, by chromosome.

Supplement 6

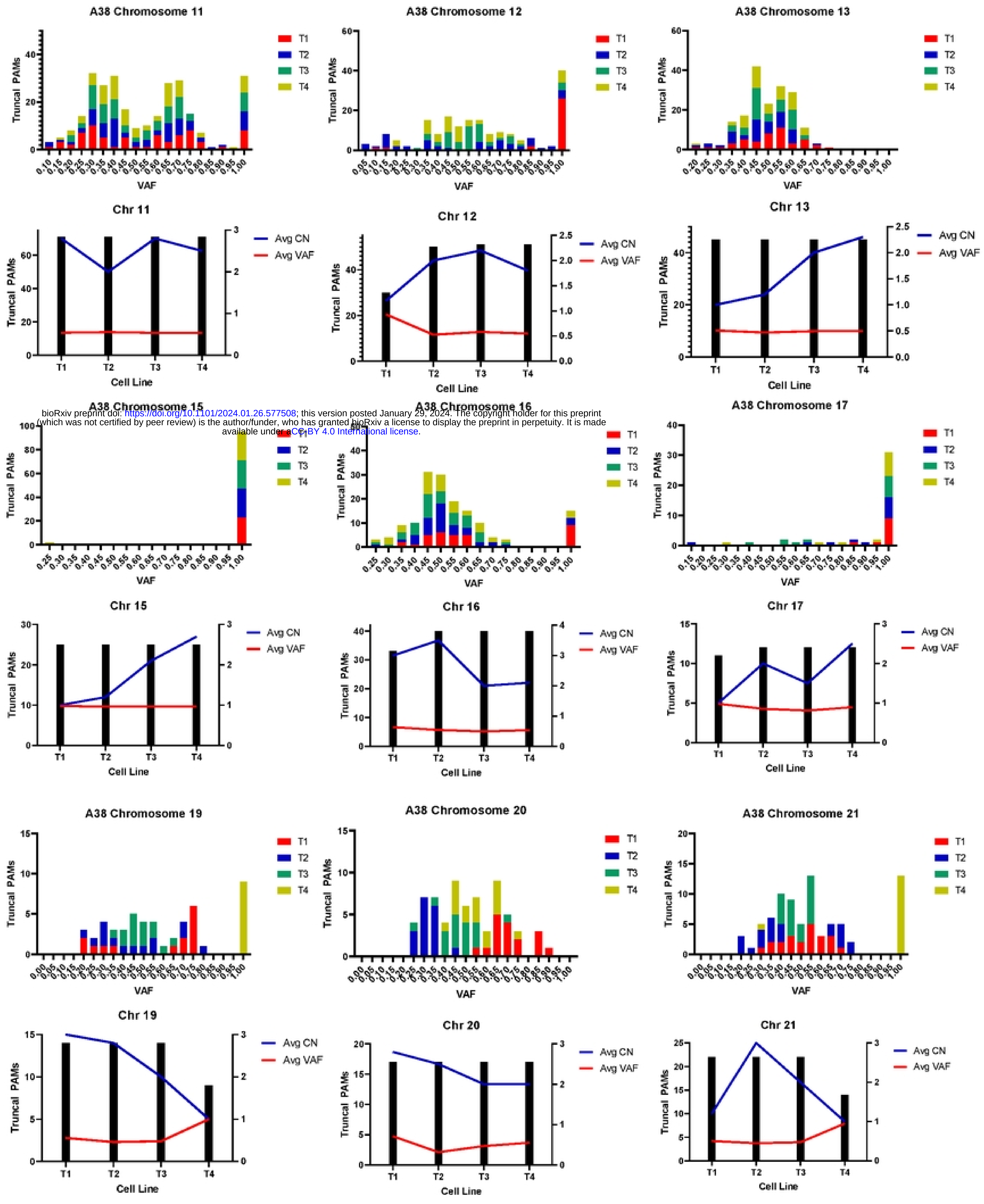

bioRxiv preprint doi: https://doi.org/10.1101/2024.01.26.577508; this version posted January 29, 2024. The copyright holder for this preprint (which was not certified by peer review) is the author/funder, who has granted bioRxiv a license to display the preprint in perpetuity. It is made available under aCC-BY 4.0 International license.

Supplemental Figure 6: Histograms and CN/VAF analysis for truncal PAMs in A38 WGS cell line data, by chromosome.

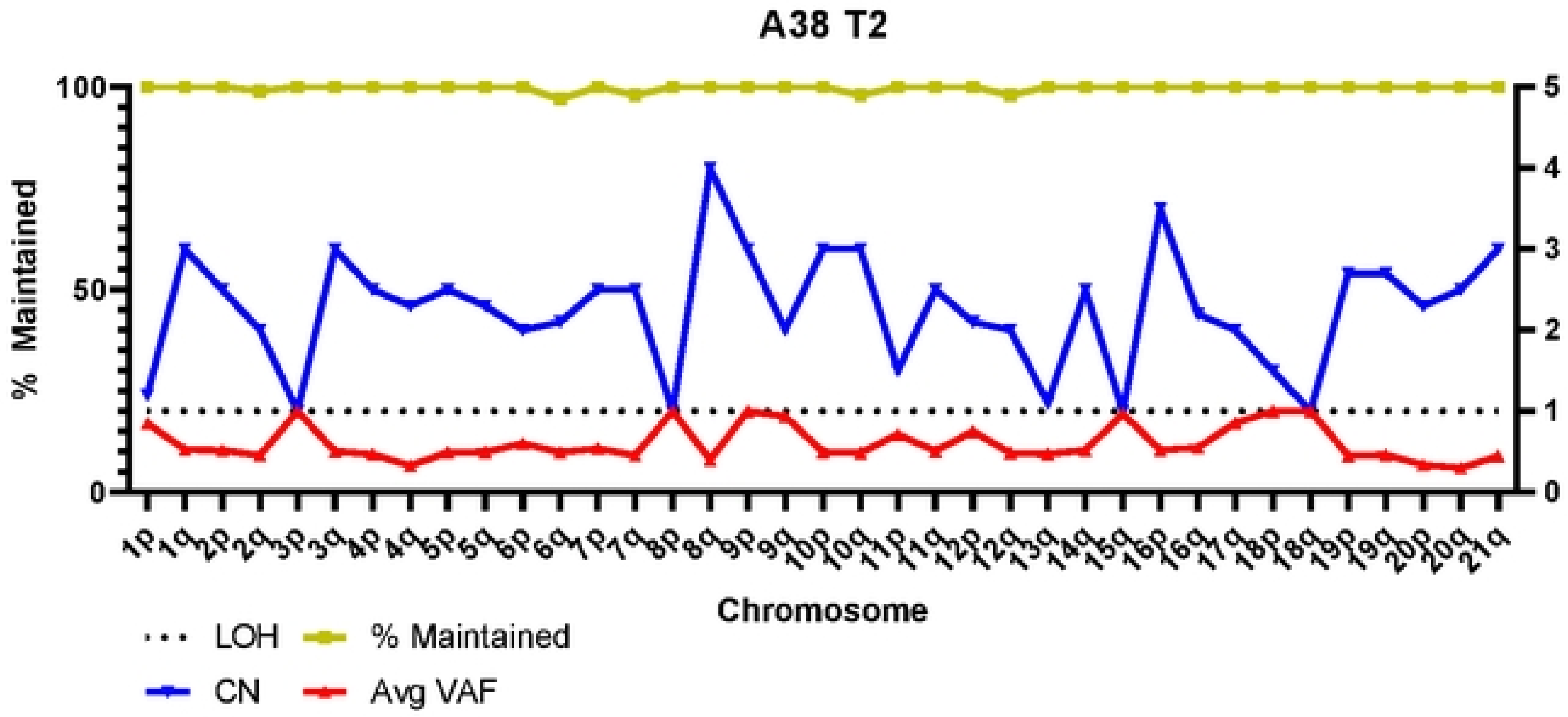

bioRxiv preprint doi: https://doi.org/10.1101/2024.01.26.577508; this version posted January 29, 2024. The copyright holder for this preprint (which was not certified by peer review) is the author/funder, who has granted bioRxiv a license to display the preprint in perpetuity. It is made available under aCC-BY 4.0 International license.

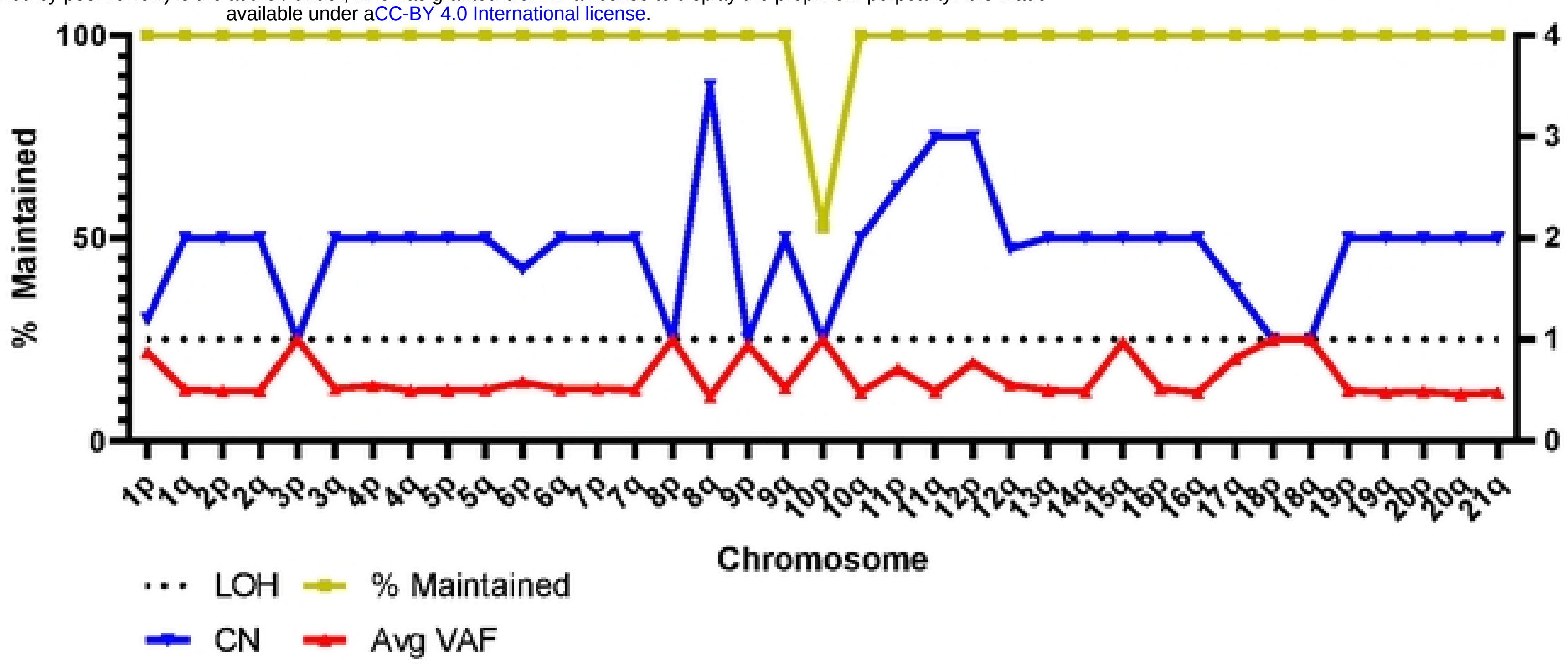

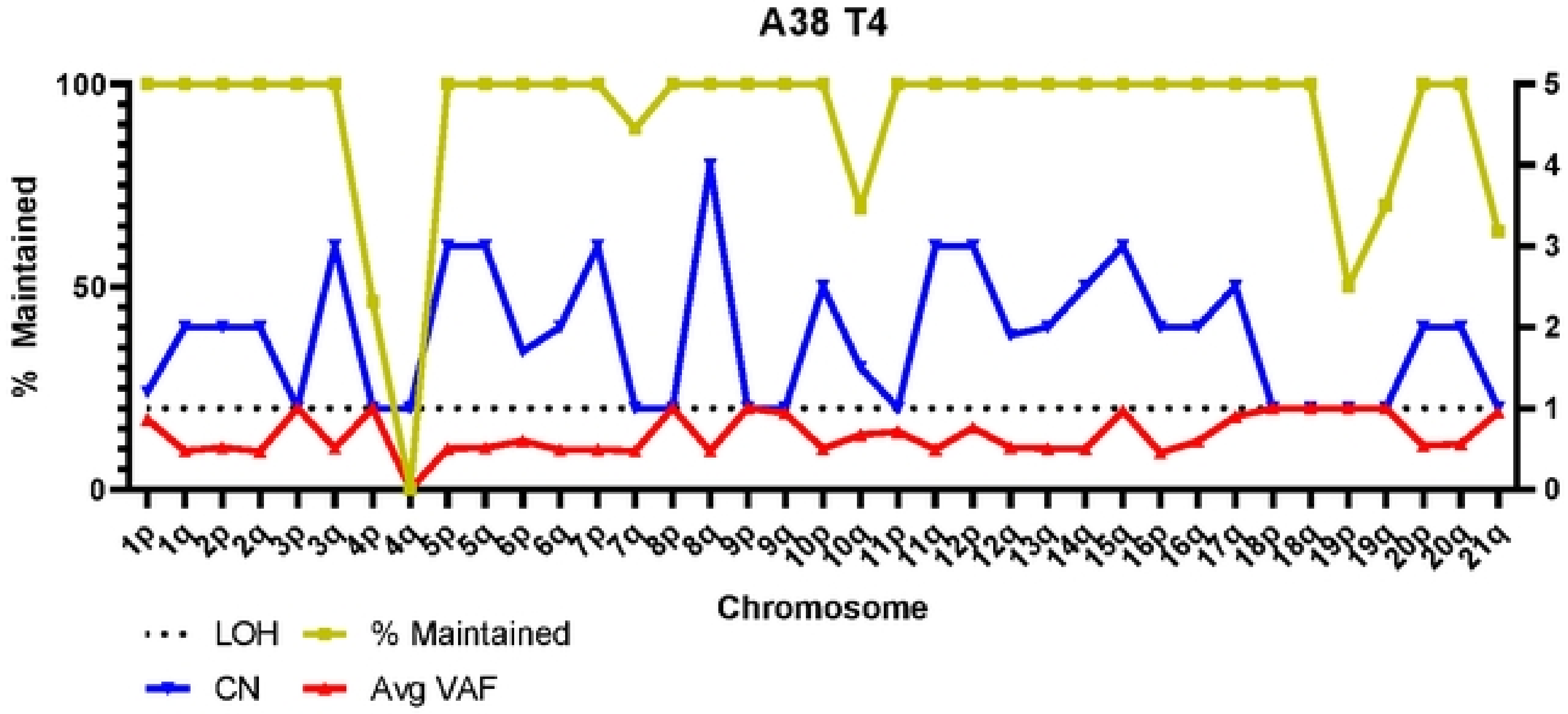

Supplemental Figure 7: Plots of PAM maintenance, VAF, and CN for A38 cell lines T2-T4 analyzed for each chromosome arm.

bioRxiv preprint doi: https://doi.org/10.1101/2024.01.26.577508; this version posted January 29, 2024. The copyright holder for this preprint (which was not certified by peer review) is the author/funder, who has granted bioRxiv a license to display the preprint in perpetuity. It is made available under aCC-BY 4.0 International license.

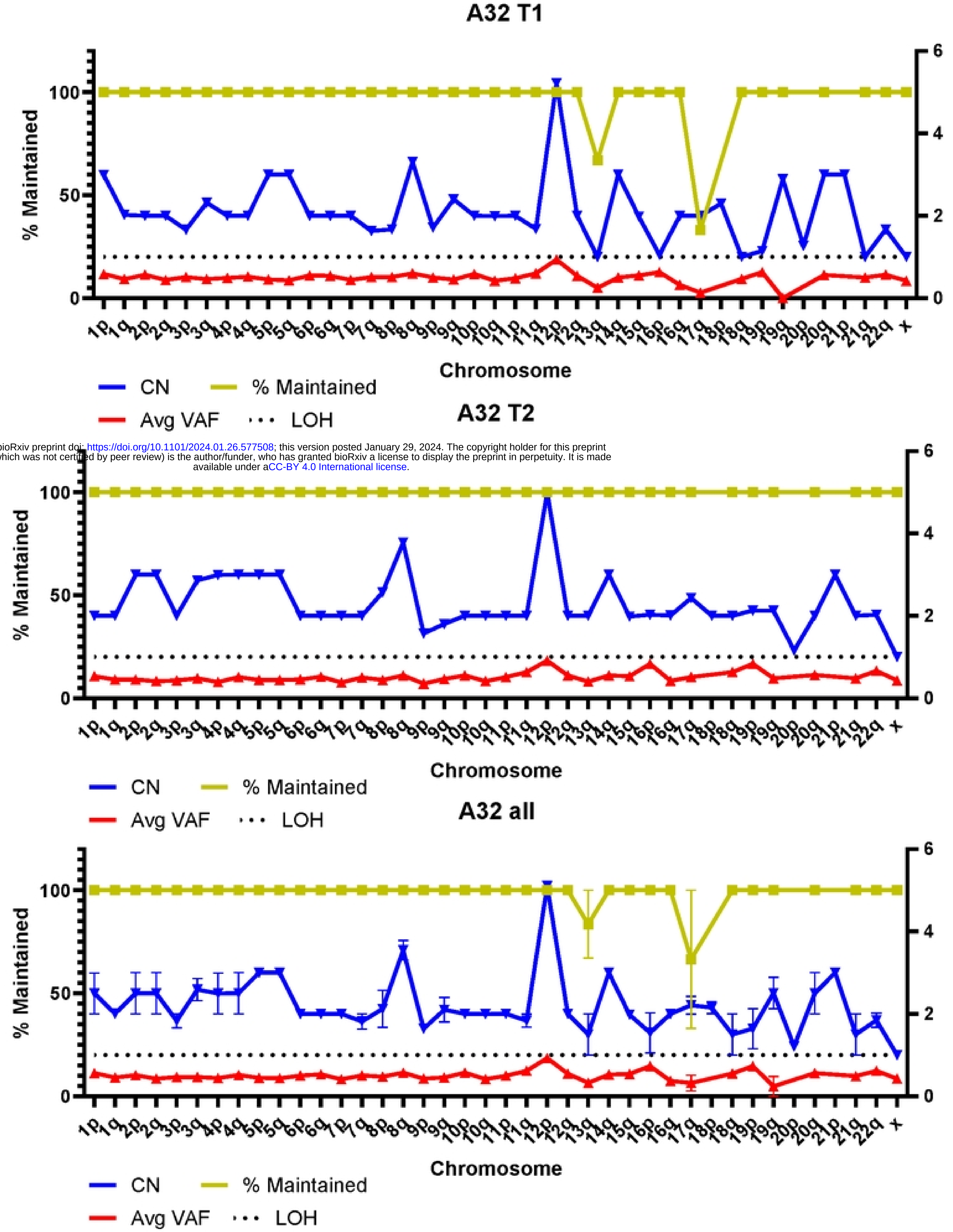

Supplemental Figure 8: Plots of PAM maintenance, VAF, and CN for A32 cell lines T1-2 analyzed for each chromosome arm.

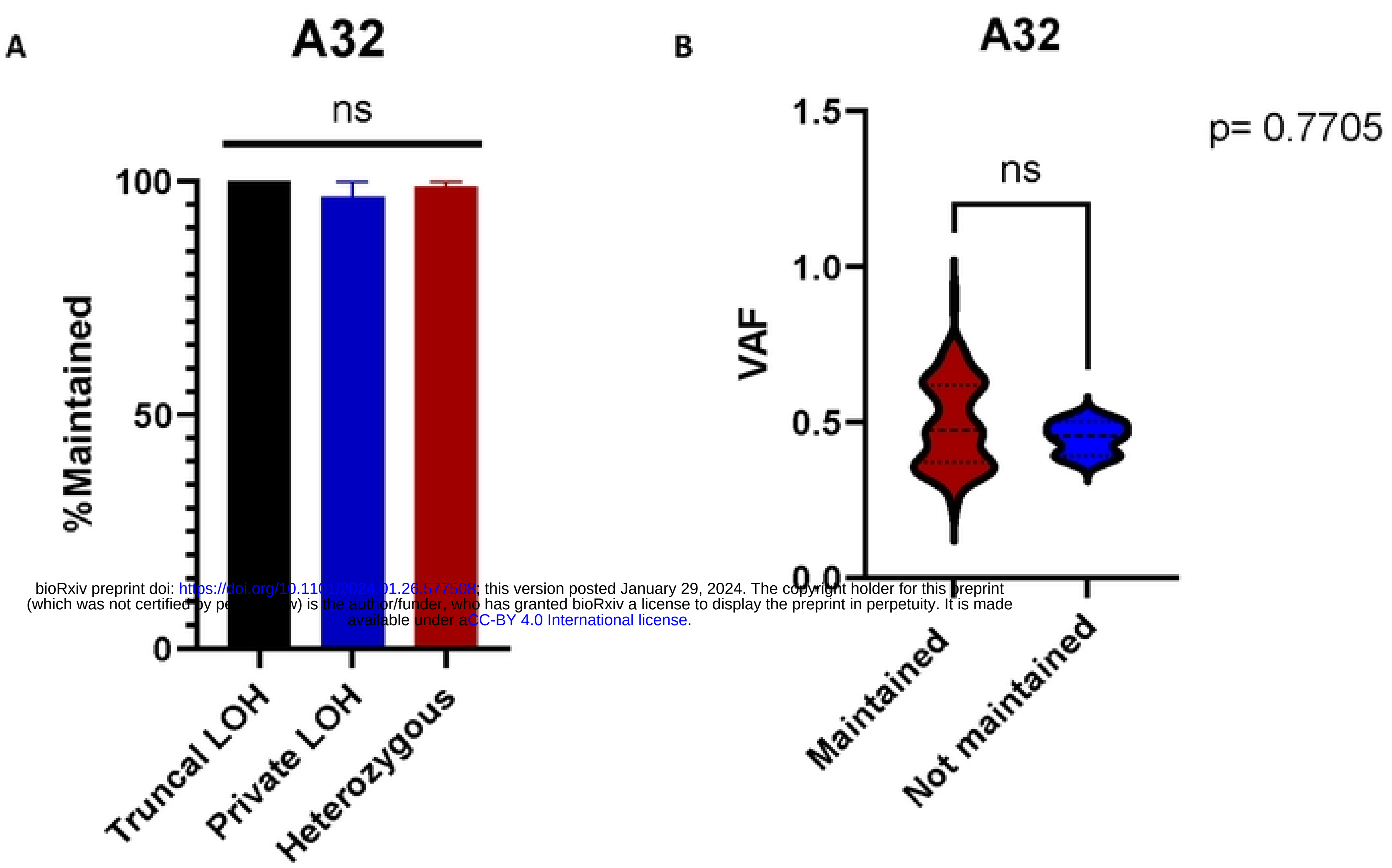

bioRxiv preprint doi: https://doi.org/10.1101/2024.01.26.577508; this version posted January 29, 2024. The copyright holder for this preprint (which was not certified by peer review) is the author/funder, who has granted bioRxiv a license to display the preprint in perpetuity. It is made available under aCC-BY 4.0 International license.

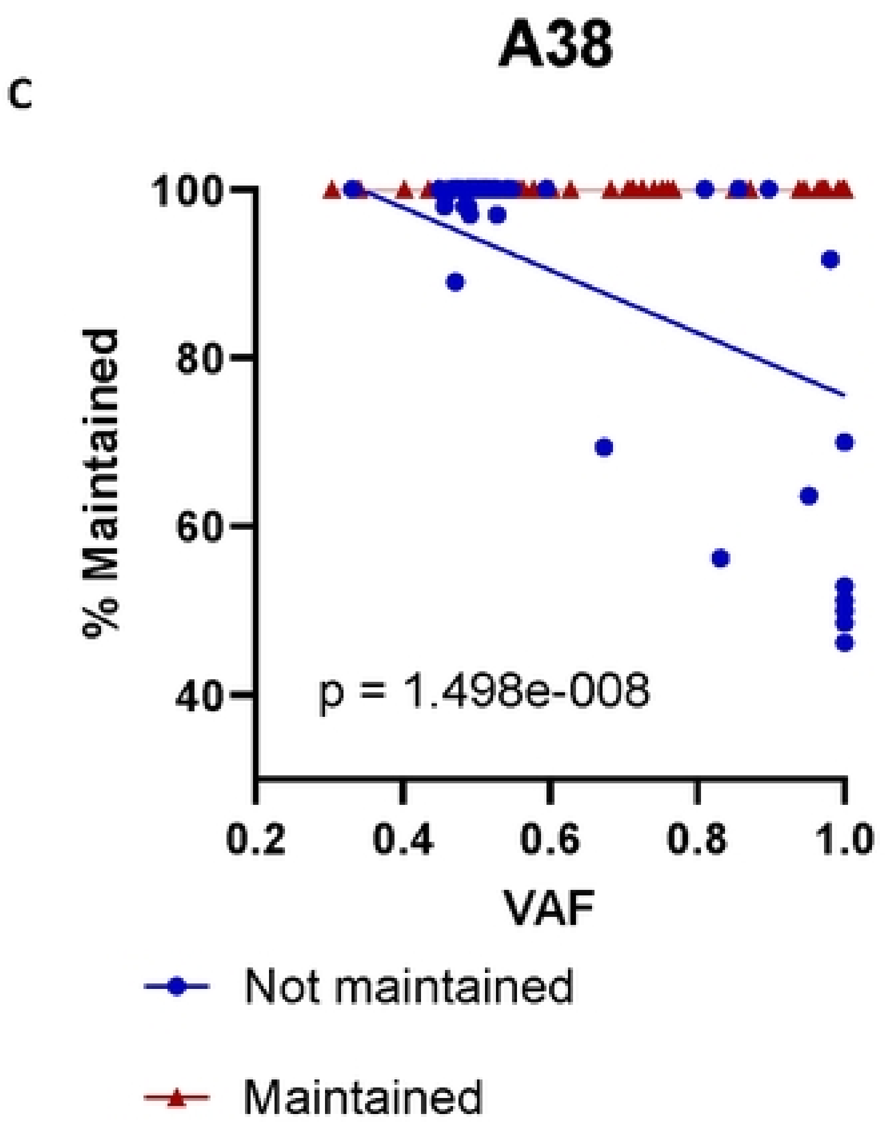

Supplemental Figure 9: WGS was available for 2 cell lines and normal for case A32. **A)** No significant difference was found between the two cell lines for LOH and PAM maintenance (Kruskal-Wallis, p=0.3307) or for **B)** VAF between truncal PAMs maintained in both lines or lost in some (Mann-Whitney rank sum, p = 0.7705). n=165 for maintained PAMs, n=3 for PAMs lost in one cell line but not the other. **C)** Plot of truncal PAM VAF comparing PAMs maintained in all four cell lines (blue) and truncal PAMs lost in at least 1 cell line (red). A wide VAF distribution in present in PAMs present in all. For PAMs lost in some, VAFs cluster at 0.5 in samples where they are maintained, 1 when they are lost at a rate of 50%.
